# Super-twisting MPPT control for grid-connected PV/battery system using higher order sliding mode observer

**DOI:** 10.1038/s41598-024-67083-w

**Published:** 2024-07-18

**Authors:** Vijaya Kumar Dunna, Kumar Pakki Bharani Chandra, Pravat Kumar Rout, Binod Kumar Sahu, Premkumar Manoharan, Anas R. Alsoud, Bizuwork Derebew

**Affiliations:** 1 Department of Electrical Engineering, ITER, Siksha ‘O’ Anusandhan (Deemed to be University), Bhubaneswar, 751030 India; 2 Department of EECE, and Center for Autonomous Systems, GITAM (Deemed to be University), Visakhapatnam, India; 3 Department of Electrical and Electronics Engineering, ITER, Siksha ‘O’ Anusandhan (Deemed to be University), Bhubaneswar, 751030 India; 4 Department of Electrical and Electronics Engineering, Dayananda Sagar College of Engineering, Bengaluru, 560078 Karnataka India; 5 Hourani Center for Applied Scientific Research, Al-Ahliyya Amman University, Amman, Jordan; 6 Department of Statistics, College of Natural and Computational Science, Mizan-Tepi University, Tepi, Bushira Ethiopia

**Keywords:** Electrical and electronic engineering, Mathematics and computing

## Abstract

In recent times, photovoltaic (PV) power generation has been growing due to increase in energy demand. In grid-connected mode, achieving maximum power (MP) from the PV array is difficult by using conventional techniques due to various reasons like low tracking efficiency, stability issues, etc. This motivates the design of an appropriate control strategy to obtain the maximum power point tracking (MPPT) to harvest MP from the PV array. This paper proposes a combined higher order sliding mode observer (HOSMO)–super-twisting control (STC) for a grid-connected scenario. A perturb and observe (P &O) technique is employed to generate reference voltage, and a HOSMO is proposed to drive the STC by estimating the inductor current of the PV boost converter. The proposed controller performance is evaluated based on response time across various scenarios, including generation changes, dynamic faults, islanding and resynchronization, and load variations in comparison to other existing controllers. These microgrid test cases have been thoroughly simulated, and their effectiveness has been validated in real-time using OPAL-RT (OP4510).

## Introduction

To meet the high electricity demand, bulk power is currently being generated at the distribution level by renewable energy sources (RES) integration. However, very often, in adverse situations, environmental concerns are taken into account to create clean energy without relying on fossil fuels. As a result, annual PV growth increased by 60% from 2004 to 2011, with annual generation capacity exceeding 200 GW in 2019^1^. According to the 2022 global status report from the Renewable Energy Policy Network for the 21st Century (REN21) highlights a remarkable increase in global renewable energy capacity over the past ten years. Solar photovoltaic (PV) installations, wind farms, and hydroelectric dams are now prevalent in landscapes, including the deserts of India^2^. By the end of 2021, the worldwide installed capacity of RES reached approximately 3146 GW. This total includes 1195 GW from hydro power, 942 GW from photovoltaic (PV) systems, 845 GW from wind energy, 143 GW from biomass power, 14.5 GW from geothermal energy, 6 GW from concentrating solar thermal power, and 0.5 GW from ocean power. The photovoltaic systems have experienced the most rapid growth between 2016 and 2021. During this period, the installed capacity of PV systems surged from 305 to 942 GW^3^. This indicates not only for the present situation but also for the future; renewable energy generation is becoming a huge revolution in smart-grid and microgrids. However, the focus of microgrid research has shifted towards integrating battery energy storage systems (BESS) into the distributed generation (DG) based system to enhance the stability and operational performance of the overall system. In the grid-connected mode, an energy storage system is necessary for microgrid operation to supply the power demand between generation and distribution^4^ for better power regulation. Many authors have proposed various control techniques for microgrid, etc. In^5^, an evolutionary BAT-Fuzzy controller for supplying continuous power to the DC load and to the grid simultaneously. A model predictive control technique for PV-battery-based microgrids is suggested in^6,7^. Recently, a direct duty-cycle controller based MPPT has been proposed in^8^. There are numerous challenges include inadequate technical infrastructure could strain power networks during large-scale integration. Additionally, the lack of dispatchability of grid connected photovoltaic system complicates grid management^9^. Moreover, the grid poses compatibility and stability concerns due to the volatile output of solar power. The unpredictable output of grid connected photovoltaic system may prompt stricter regulations regarding grid interconnection by utilities and system operators. Another challenge is the limited contribution of this system during peak demand periods, leading to operational complexities along with MPPT control^10^.

The PV array’s ability to generate electricity is mostly determined by the weather conditions (irradiance and temperature). As a result, to collect the MP from a PV array and enhance system efficiency, an MPPT technique is required^8^. Several MPPT algorithms have been explored by the researchers over the last few decades to improve performance and achieve faster convergence^11^; preferred techniques include Perturb and Observe (P &O) method, Incremental Conductance (IC) approach, and Hill-Climbing (HC)^12,13^. In^14^, the authors identified that these control methods are not efficient in grid connected mode of operation because of oscillations around the MPP. Thus, further improvements by the Artificial Neural Networks (ANN) and Fuzzy logic (FLC) based control solutions are introduced in^15–17^. To implement these control methods, significant data sets based on real-time characteristics of PV array are required, which makes the design procedure more complex. Because of these reasons, the control system takes a longer time to execute the algorithm, and it is difficult to ensure better efficiency in real-time context^11^. Many researchers are exploring robust control strategies for better performance and efficiency.

The robust control provides great dependability and stability in the system’s unpredictability response. Sliding mode control (SMC) is one of the most often used techniques in robust control^18^ for various engineering applications to handle uncertainties and to provide precise tracking and robustness towards disturbances and inaccuracies^19^. However, it is a well accepted fact that, in general, the conventional SMC is prone to high frequency chattering, which will impact the plant’s output voltage response. A super-twisting algorithm-based second order SMC is introduced in^20^ to eliminate the chattering effect and capture the maximum power from the PV array. However, in exceptional cases, the STC utilizes only the upper bound of uncertainty, which is one of the intrinsic weaknesses of STC^18^. To improve accuracy in PV systems output under MPPT conditions, a robust observer-based FLC and back-stepping control strategies are described in^21,22^ to estimate disturbance entering the system. To get chatter-free output voltage from the PV system,^23^ introduces MPPT based on a second-order sliding mode gradient observer-based control. However, sometimes the second-order observers estimate the incorrect values because of unidentified uncertainty caused by the source. This inaccurate estimation affects the steady-state accuracy of MPPT control signal^18^. The HOSMO based integral sliding mode control (ISMC) is presented in^24^, but the ISMC could not able to provide finite time convergence and is a discontinuous controller^25^. These issues frame the objectives of the study to provide an advanced HOSMO-STC that takes these difficulties into account to achieve MPPT under different conditions and variations in the grid connected mode of operation.

This paper’s contribution to the field of research is summarised as follows:

1. Design of HOSMO to estimate the inductor current for a micro-grid
2. Super-twisting controller design to track the MP of microgrid accurately, which also ensures the system’s finite-time stability
3. Design of combined HOSMO based super-twisting controller for a microgrid using a limited number of outputs
4. Comparison of the proposed HOSMO based super-twisting controller with controller based on extended Kalman filter and super-twisting observer for a microgrid
5. Real-time simulations using OPAL-RT to verify the efficacy of the proposed controller-observer scheme on a microgrid under various test conditions

The rest part of the paper organizes into seven sections. Section “Proposed dc microgrid system” describes the complete structure of the grid-connected PV battery system with the mathematical modeling of the PV array, boost converter, and battery energy storage. Section “Proposed super-twisting controller based on HOSMO for a microgrid” demonstrates the complete modeling of conventional approaches used for MPPT control, such as P &O and STC. In addition to this, HOSMO-super-twisting controller design and its stability proof are presented. A detailed description of the OPAL-RT experiment and its hardware setup is presented in “Implementation of the proposed system on OPAL-RT”. The results and discussion for different test cases are illustrated and analyzed in “Results and discussion”. Finally, “Conclusions” concludes the study, followed by relevant references.

## Proposed dc microgrid system

**Figure 1:**
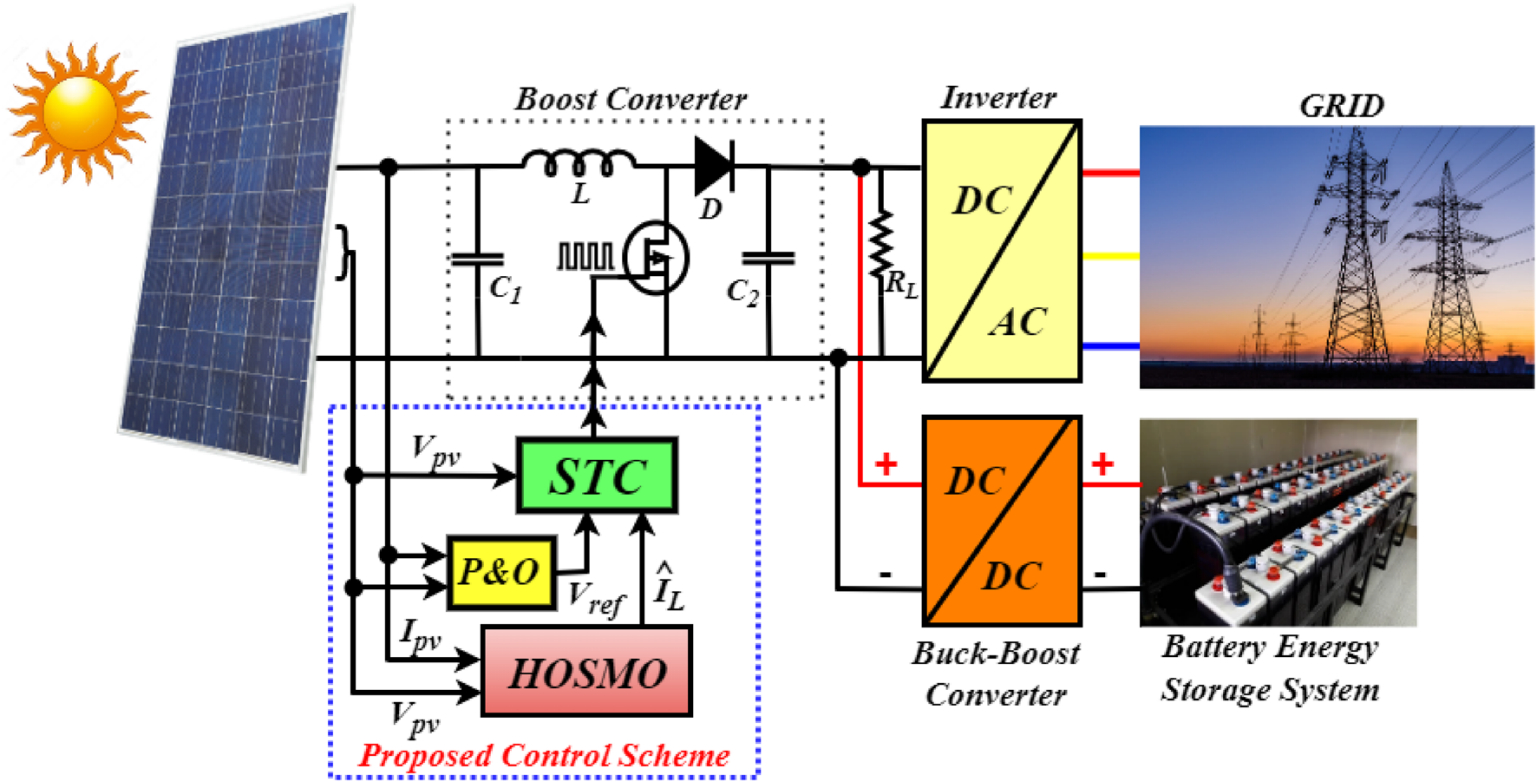
Proposed combined HOSMO based STC for PV/battery system.

The grid-connected PV-Battery system with the proposed controller-observer is shown in Fig. 1. The PV array is connected to a DC–DC boost converter to generate the required power from the PV, and the corresponding gating pulses are generated using the proposed control scheme. A Lithium ion based Battery Energy Storage System (BESS) is connected through a bidirectional DC–DC buck-boost converter, operated as a secondary source in the system to regulate the power at DC load. BESS is operated with a charging-discharging control strategy to make the system feasible and realistic for real-time operation. The utility grid is interfaced at the Point of common coupling (PCC) through an IGBT inverter. The control operation of the inverter is accomplished by the proportional resonant (PR) controller detailed^26^ and^27^. Zero level control in inverter operation precisely refers to the regulation of the inverter output to achieve perfect synchronization with the voltage waveform of the utility grid. This synchronization ensures seamless integration between the inverter and the grid, minimizing disruptions and optimizing power transfer efficiency. These controllers divided into current and voltage based controllers. The first level of hierarchical control in a system, referred to as primary control or local control is characterized by its rapid response capability. Its main objective is to maintain voltage and frequency stability within Microgrids (MG) by efficiently managing load distribution among Distributed Generation (DG) units^28^.

**Figure 2:**
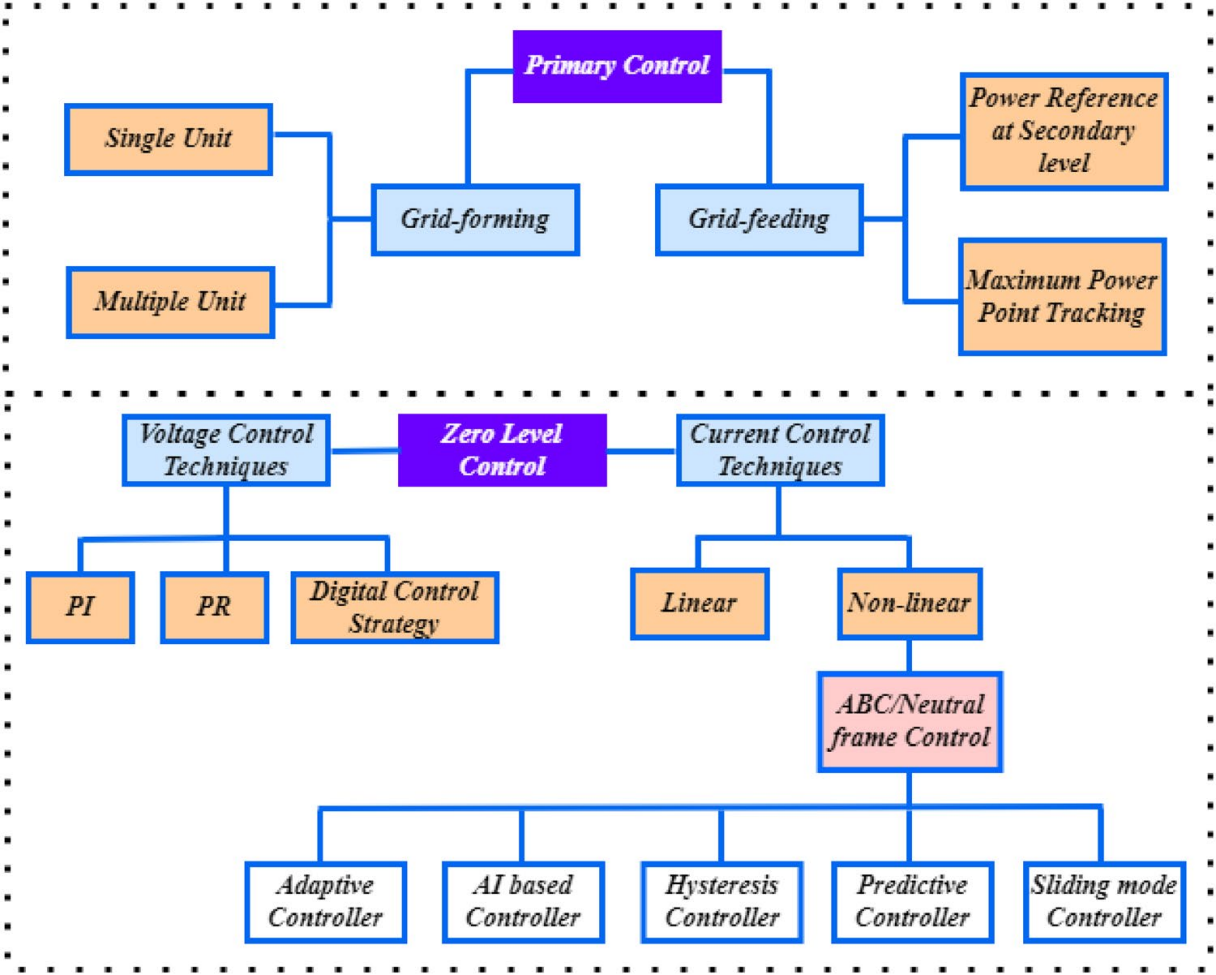
Zero level and primary controllers classification^28^.

In the primary control level, communication-based control offers fast dynamics control, enhanced power sharing, and improved voltage/frequency (V/f) quality. However, this approach sacrifices system flexibility and is susceptible to interruptions in communication lines. As an alternative, wireless communication-based droop control is emerging as a promising and trending solution. Despite facing some drawbacks, ongoing efforts are focused on refining various droop-based control techniques to address these challenges. This can be explained in Fig. 2.

The conventional approach based on the P &O algorithm is applied in the proposed control system to generate the reference voltage $V_{ref}$. The key purpose of the HOSMO is to accurately estimate the inductor current $I_{L}$ based on measurements of PV voltage $\left( V_{pv}\right)$ and PV current $\left( I_{pv}\right)$. The STC provides duty ratio ($\textit{d}$) and ($\textit{1-d}$) to the switch of the boost converter for turn-on and turn-off operation, which makes the PV operational performance more effective. The objective of this work is to provide continuous power to the utility and protects the system without affecting the power supply reliability and regulation performance using the proposed HOSMO based super-twisting controller. In the next subsections, each source characteristics and their contribution are explained.

### Photovoltaic modelling

**Figure 3:**
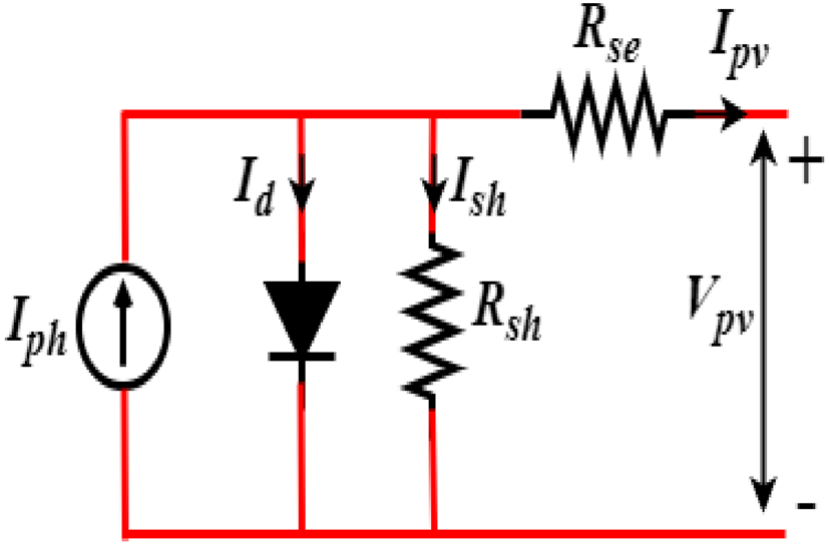
Single diode PV circuit.

PV arrays are the composition of series and parallel cells of PV panels to generate DC power from solar energy. The mathematical expression of PV current for the corresponding single diode PV model is obtained from the circuit diagram presented in Fig. 3^11^.

1 $$\begin{aligned} \left\{ \begin{array}{l} I_d = I_{ph} - I_{sh} \left( \exp \left( \frac{q(V_{pv}-R_{se})}{N_s KT} \right) - 1 \right) - \frac{V_{pv} + R_{se}}{R_{sh}} \\ I_{ph} = \frac{G}{G_{ref}} \left( I_{pvn} + K_i (T - T_{ref}) \right) \\ I_{sh} = \frac{I_{sat} + K_i (T - T_{ref})}{\exp \left( \frac{V_{oc} + (K_i (T - T_{ref}))}{A(N_s KT)} \right) - 1} \end{array} \right. \end{aligned}$$

where $I_{ph}, I_{d}$ and $I_{sh}$ are photo current, diode current, and shunt current, respectively, $I_{sat}$ is the reverse saturation current, *G* and $G_{ref}$ are solar irradiance and its reference values in $W/m^{2}$, *T* and $T_{ref}$ are PV cell temperature and its reference conditions in ${}^{0}C$, $I_{scr}$ and $k_{i}$ are the PV cell short circuit current and its temperature coefficient. The $I_{pv}$ can be written as follows^29^:

2 $$\begin{aligned} I_{pv} = I_{ph} - I_{sat} N_p \left[ \exp \left( \frac{(V_{pv} + R_{se} \frac{N_s}{N_p}I_d)}{V_t AN_s} \right) - 1 \right] - \frac{(V_{pv} + R_{se} \frac{N_s}{N_p}I_d)}{R_{sh} \frac{N_s}{N_p}} \end{aligned}$$

where, $N_{p}$ and $N_{s}$ denotes the number of parallel and series cells; $R_{sh}$ and $R_{se}$ parallel and series resistances, respectively. Further, *A* is the ideality factor, *q* represents electronic charge, the Boltzmann’s constant is represented by *K*, and $V_{t}$ is the thermal voltage.

#### Boost converter modelling

The DC–DC boost converter is adopted to adjust the output voltage of the PV array to maximize the power generation from solar energy. The boost converter needs to operate with a high-frequency switching signal to achieve MPPT from the PV array. The continuous mode state-space average model of the DC–DC boost converter is obtained by applying the node equation to the boost converter circuit presented in Fig. 1. The boost converter has two different operating modes, one is ON, and another is OFF, depending on the switch position^30^.

${{\varvec{Position:1}}}$ When the switch is ON, the voltage across the switch becomes zero, and the diode is in conduction mode. The dynamics of $V_{pv}$ and $I_{L}$ can be derived as follows:

3 $$\begin{aligned} \left. \ \begin{array}{l} L\frac{dI_{L}}{dt}= V_{pv} \\ C\frac{dV_{pv}}{dt}= I_{pv}-I_{L} \end{array} \right\} ON \end{aligned}$$

${{\varvec{Position:2}}}$ When the switch is OFF, the voltage across the diode becomes zero. The dynamics of the converter can be represented as follows:

4 $$\begin{aligned} \left. \ \begin{array}{l} L\frac{dI_{L}}{dt}= V_{pv} -V_{dc} \\ C\frac{dV_{pv}}{dt}= I_{pv}-I_{L} \end{array} \right\} OFF \end{aligned}$$

The state equations of the boost converter are:

5 $$\begin{aligned} \left. \begin{array}{c} {{\dot{x}}}_1 ={{\dot{V}}}_{pv}=\frac{1}{C}\left[ I_{pv}-\delta I_{L}\right] \\ {{\dot{x}}}_2 =\ {{\dot{I}}}_L=\frac{1}{L}\left[ \delta V_{pv}- V_{dc}\right] \end{array} \right\} \end{aligned}$$

where $\delta \epsilon \left[ 0,1\right]$ is the duty cycle. $V_{pv}$ and $I_{pv}$ are the input voltage and current, respectively. $I_{L}$ and $V_{dc}$ are the inductor current and output voltage respectively. *C* and *L* are capacitor and inductor components of the boost converter, respectively. The absence of a boost converter in single-stage architecture reduces system losses and overall costs by simplifying the design, lowering component count, and improving efficiency. This streamlined approach minimizes energy losses associated with conversion processes, decreases manufacturing and maintenance expenses, and enhances system reliability, resulting in significant cost savings over the system’s lifetime^31^. The control signal of the boost converter is generated by the control system proposed in this paper, which will be discussed in the next section.

### Battery energy storage system (BESS)

**Figure 4:**
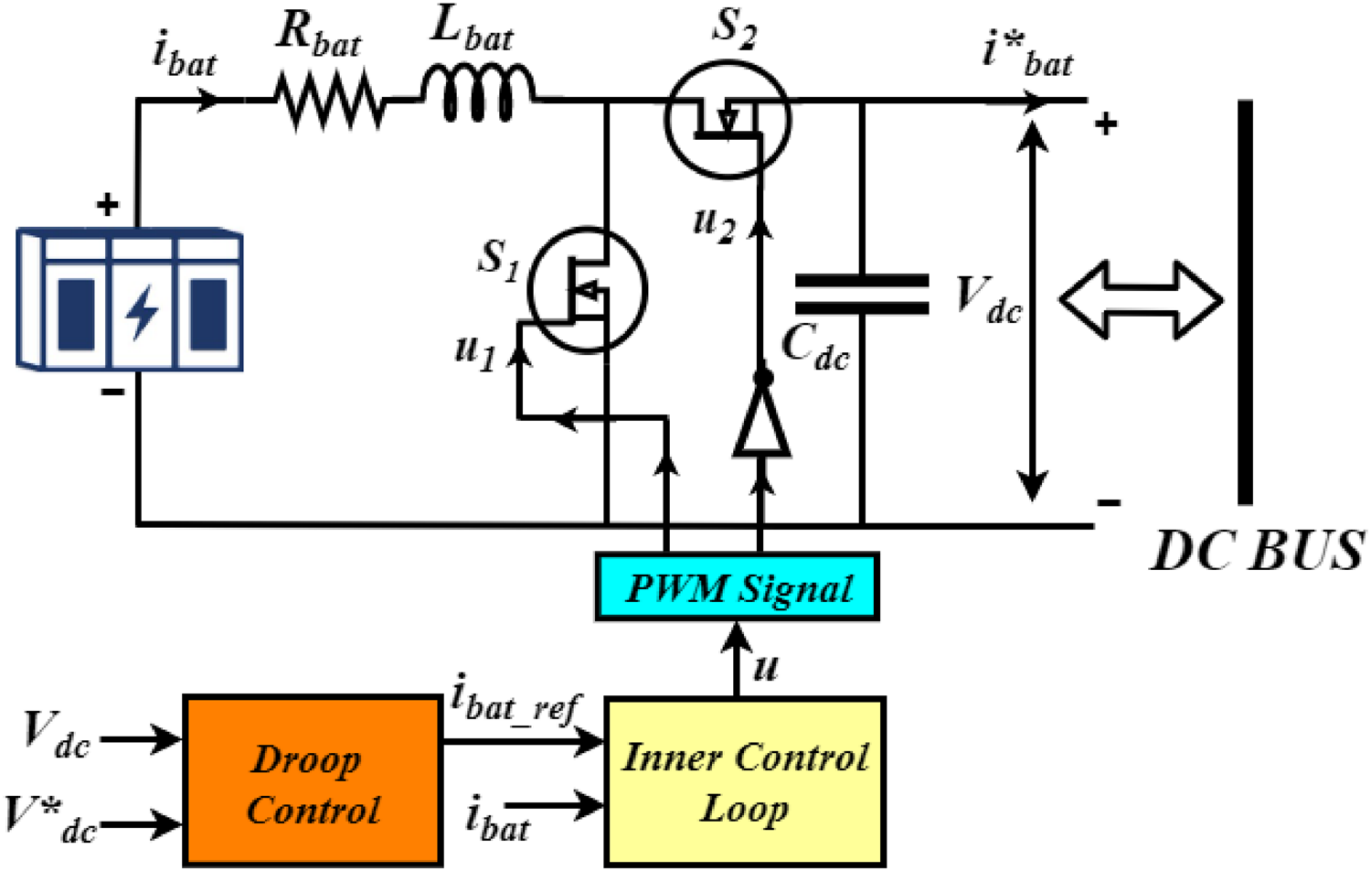
BES system and its control strategy.

Figure 1 demonstrates the connection of BESS with the PV array to enhance the power regulation to meet the power demand required for the load and grid. BESS is essential for integrating renewable energy, stabilizing the grid, reducing peak demand, providing backup power, enabling energy arbitrage, supporting electrification, decentralization, market participation, environmental benefits, and cost savings. In the grid connected mode the generation is greater than the load in standalone mode battery is charging and the battery converter system is operated in buck mode. In off-grid mode, battery storage systems discharge power when the demand exceeds the available generation^32^. The buck-boost converter has an inductor $L_{bat}$, an equivalent resistance $R_{bat}$, an output filter capacitor $C_{dc}$, and two switches $S_{1}$ and $S_{2}$ as demonstrated in Fig. 4. The droop control strategy is adopted for the correct and optimal PWM signal generation and to control both the associated switches accordingly. The proposed control approach regulates the charging and discharging mode of operation according to the state of charge (SoC) and power demand at the load side. The discharging mode of operation (Boost mode) of the battery has occurred under the $S_{1}$ switch at ON state and $S_{2}$ at OFF state condition, and the battery delivers the required amount of power to the DC bus during this period. Similarly, the charging mode of operation (Buck mode) has occurred under the $S_{1}$ is at OFF condition and $S_{2}$ is at ON condition, and the battery is charged by receiving power from the DC grid. The charging and discharging modes are mathematically presented as follows:

6 $$\begin{aligned} \ u = \left\{ \begin{matrix} 1\ if\ \left( i_{bat\_ref}>0\right) \\ 0\ if\ \left( i_{bat\_ref}<0\right) \\ \end{matrix} \right. \ \end{aligned}$$

where, $i_{bat\_ref}$ is the reference current of the battery computed and generated by applying the droop control strategy^33^. The DC bus voltage droop concept adopted in this study can be expressed as follows:

7 $$\begin{aligned} i_{d} = -k_{d}\times \left( {V_{dc}}^{*} - V_{dc} \right) \end{aligned}$$

where, $k_{d}$ is the droop coefficient, ${V_{dc}}^{*}$ is the nominal DC bus voltage, and $V_{dc}$ is the DC bus voltage.

The battery converter dynamics in both boost mode and buck mode of operation as presented in^34^, can be simplified as follows:

8 $$\begin{aligned} \frac{di_{bat}}{dt}= & {} \frac{V_{bat}}{L_{bat}}-\frac{R_{bat}}{L_{bat}}i_{bat}-u \frac{V_{dc}}{L_{bat}} \end{aligned}$$

9 $$\begin{aligned} \frac{dV_{dc}}{dt}= & {} u \frac{i_{bat}}{C_{dc}}-\frac{{i^{*}}_{bat}}{C_{dc}} \end{aligned}$$

where, *u* is the control signal from the droop control strategy.

## Proposed super-twisting controller based on HOSMO for a microgrid

### MPPT by the P &O algorithm

The P &O based MPPT control is applied to track MPP in the PV cell. This conventional approach is considered in this study due to its extensive real-time applications in power systems, easy implementation, less complex design, and cost-effectiveness. The reference voltage signal for the proposed approach is computed under variable environmental conditions like solar irradiance and temperature by the P &O method. When the operating point exists on the left side of the MPP, the power increases with the voltage increase. On the other hand, when the operating point exists on the right side of the MPP, the power decreases with the voltage increase. The algorithm is repeatedly followed in this method with the two basic principles until the power reaches the maximum point^35^. Under the sudden climatic change, this algorithm takes a larger time to reach the optimal MPP to cope with and sometimes leads to oscillations around the optimal point of power extraction. To handle this issue, many suggestions have been made in recent times by many authors. However, the low tracking efficiency of the P &O still is a major issue and is considered as a major limitation, particularly under complex conditions, which needs to focus further. To handle this issue, an advanced control strategy STC based on HOSMO is proposed to improve the accuracy and efficiency of the MPPT control.

### Design of Super-twisting Controller (STC)

In this work a sliding mode controller based on the super-twisting algorithm is employed, the primary benefit of using a sliding mode controller based on the super-twisting algorithm is that it retains all the characteristics of the conventional sliding mode control while suppressing the jitter phenomenon. Additionally, its trajectory in the sliding plane is twisted around the origin^36^. The twisting algorithms is designed for smooth systems, offer the capability to replace discontinuous control with absolutely continuous control. As compared with the other sliding mode controllers the proposed controller is allowing for precise compensation of absolutely continuous disturbances, which is shown in Table 1. However, to address chattering, a phenomenon marked by rapid and excessive control oscillations, the controller must incorporate chattering attenuation techniques^18,25^.

**Table 1:** Comparison of sliding mode controllers.

Type of controller	Type of convergence	Output tracking	Description
Conventional SMC	Finite time convergence	Asymptotic	Discontinuous
Integral SMC	Asymptotic convergence	Asymptotic	Discontinuous
Super-twisting SMC	Finite time convergence	Asymptotic	Continuous

The STC comprises two parts (equivalent control and switching control) and is given as follows:

10 $$\begin{aligned} u\left( t \right) = u_{con}+u_{dis} \end{aligned}$$

where, $u_{con}$ is the continuous control operated in the obscene of external disturbance and uncertainty. The $u_{dis}$ is the discontinuous control signal that acts when external disturbance and uncertainty enter the system. The function of the control system is to obtain the switching control signals for the boost converter. The optimal duty cycle is computed within $0 \le u \le 1$ during the system operation. The switching sequence of the boost converter is:

11 $$\begin{aligned} \ u = \left\{ \begin{matrix} 1\ when\ S < 0 \\ 0\ when\ S > 0 \\ \end{matrix} \right. \ \end{aligned}$$

The sliding surface *S* is considered as follows:

12 $$\begin{aligned} S=\sigma e+{\dot{e}} \end{aligned}$$

where, $\sigma$ is the sliding coefficient and the error *e* is given as follows:

13 $$\begin{aligned} e =V_{ref}-V_{pv} \end{aligned}$$

where, $V_{ref}$ is the desired value generated from the P &O algorithm and $V_{pv}$ is the actual PV voltage from the PV array. The derivative of the sliding surface is:

14 $$\begin{aligned} {\dot{S}}=\sigma {\dot{e}}+\ddot{e} = \sigma \left( {{\dot{V}}}_{ref}-{{\dot{V}}}_{pv}\right) +{{\ddot{V}}}_{ref}-{{\ddot{V}}}_{pv}. \end{aligned}$$

To get the continuous control input, Eq. (5) is substituted in Eq. (14), and setting the ${\dot{S}}=0$, the $u_{con}$ is derived as follows:

15 $$\begin{aligned} 0= & {} \sigma \left( {{\dot{V}}}_{ref} -\frac{I_{pv}}{C}-\frac{I_{L}}{C} u_{con} \right) +{{\ddot{V}}}_{ref}-{{\ddot{V}}}_{pv} \end{aligned}$$

16 $$\begin{aligned} u_{con}= & {} \frac{C}{I_{L}}\left[ \frac{{{\ddot{V}}}_{ref}}{\sigma } - \frac{{{\ddot{V}}}_{pv}}{\sigma }+{{\dot{V}}}_{ref}-\frac{I_{pv}}{C}\right] \end{aligned}$$

The final continuous control is written as follows:

17 $$\begin{aligned} u_{con}= \frac{C}{I_{L}} \left[ \frac{1}{\sigma }\left\{ \frac{d}{dt}\left( \frac{dV_{ref}}{dt}-\frac{dV_{pv}}{dt}\right) \right\} +\frac{dV_{ref}}{dt}-\frac{I_{pv}}{C}\right] \end{aligned}$$

The $u_{dis}$ is proposed to compute according to the super-twisting algorithm^37,38^, as given below:

18 $$\begin{aligned} u_{dis}= -k_{1}{\mid S \mid }^{\frac{1}{2}} sgn\left( S \right) -\int ^{t}_{0} k_{2} sgn(S) d\tau \end{aligned}$$

where, $k_{1}$ and $k_{2}$ signifies two positive constants. From Eq. (10), the control signal can be represented as:

19 $$\begin{aligned} \begin{aligned} u(t)&= \frac{C}{I_{L}} \left[ \frac{1}{\sigma }\left\{ \frac{d}{dt}\left( \frac{dV_{ref}}{dt}-\frac{dV_{pv}}{dt}\right) \right\} +\frac{dV_{ref}}{dt}-\frac{I_{pv}}{C}\right] \\&\quad -k_{1}{\mid S \mid }^{\frac{1}{2}} sgn\left( S \right) -\int ^{t}_{0} k_{2} sgn(S) d\tau \end{aligned} \end{aligned}$$

#### Assumption 1

Here, the inductor current $I_L$ is the estimated state variable. And remaining states of the system are assumed to be accessible and measurable.

The conventional Sliding Mode Control (SMC) sliding surface *S* and its derivative ${\dot{S}}$ will drive to zero in finite time only in the presence of disturbances with known boundaries. However, the Super Twisting control algorithm can robustly handle this issue even when the disturbance boundaries are unknown. For the system in Eq. (5), there exists a range of values for the gain $\sigma$ such that the surface *S* and ${\dot{S}}$ are forced to zero in finite time and remain zero thereafter (referring Proposition 1^39^). In this work, an appropriate gain value for $\sigma$ is selected to address this problem by generating a continuous control function that attenuates chattering. This ensures that the gains adapt to unknown additive and multiplicative perturbations with unknown boundaries^40^. To design the HOSMO based super-twisting control concept, the true value ${\dot{x}}_{2}$ is replaced by its estimate and has been derived in the next section.

### Design of higher order sliding mode observer (HOSMO)

Consider the following system dynamics^23^.

20 $$\begin{aligned} \begin{array}{l} {{\dot{x}}}_{1}= f_{1}\left( x_{1},u \right) +g\left( t \right) x_{2}\\ {{\dot{x}}}_{2}= f_{2}\left( x_{1},x_{2},u \right) +\varphi \left( t \right) \end{array} \end{aligned}$$

where, $y = x_{1}$ is measured output variable, and $x_{2}$ is an unknown state to estimate, *u* is the known input, $f_{1}$ and $f_{2}$ are the known continuos and discontinuous functions. $\varphi$ represents uncertainty, and $g\left( t \right)$ is a time-varying coefficient^37,41^. The unmeasured state $x_{2}$ is estimated using HOSMO from the measurement of $x_{1}$. The estimated states are mentioned as follows^38^:

21 $$\begin{aligned} \left. \ \begin{array}{l} {\dot{{\hat{x}}}}_1= {{\hat{x}}}_2+u+z_1 \\ {\dot{{\hat{x}}}}_2= {{\hat{x}}}_3+z_2\\ {\dot{{\hat{x}}}}_3= z_3 \end{array} \right\} \end{aligned}$$

where, correlation terms are expressed as $z_{1},z_{2}$ and $z_{3}$. When the system is operated without external disruption for a specified period, then ${\dot{{\hat{x}}}}_3=0$. Now, the errors become as follows:

22 $$\begin{aligned} \begin{aligned} e_{x_{1}}&=x_{1}-{\hat{x}}_{1} \\ e_{x_{2}}&=x_{2}-{\hat{x}}_{2} \end{aligned} \end{aligned}$$

The correlation terms are computed as follows:

23 $$\begin{aligned} \left. \ \begin{array}{l} z_1= l_1{\left| e_{x_{1}}\right| }^{\frac{2}{3}}sgn\left( e_{x_{1}}\right) \\ z_2= l_2{\left| e_{x_{1}}\right| }^{\frac{1}{3}}sgn\left( e_{x_{1}}\right) \\ z_3= l_3sgn\left( e_{x_{1}}\right) \end{array} \right\} \end{aligned}$$

Here, the $l_{1},l_{2}$ and $l_{3}$ are the positive constants. The associated error dynamics are expressed as follows:

24 $$\begin{aligned} \left. \ \begin{array}{l} {{\dot{e}}}_{x_{1}}= -l_1{\left| e_{x_{1}}\right| }^{\frac{2}{3}}sgn\left( e_{x_{1}}\right) +e_{x_{2}} \\ {{\dot{e}}}_{x_{2}}= -l_2{\left| e_{x_{1}}\right| }^{\frac{1}{3}}sgn\left( e_{x_{1}}\right) -{\hat{x}}_{3}+\varphi \\ {\dot{{\hat{x}}}}_3= l_3sgn\left( e_{x_{1}}\right) \end{array} \right\} \end{aligned}$$

#### Remark 1

The $\varphi$ is considered an unknown disturbance and assumed as a Lipschitz and ${\dot{\varphi }}<\Delta$. To achieve finite stability $l_{3}>\Delta$^18^.

Then, the finalized error dynamics become as follows:

25 $$\begin{aligned} \left. \ \begin{array}{l} {{\dot{e}}}_{x_{1}}= -l_1{\left| e_{x_{1}}\right| }^{\frac{2}{3}}sgn\left( e_{x_{1}}\right) +e_{x_{2}} \\ {{\dot{e}}}_{x_{2}}= -l_2{\left| e_{x_{1}}\right| }^{\frac{1}{3}}sgn\left( e_{x_{1}}\right) +e_{x_{3}} \\ {{\dot{e}}}_{x_{3}}= -l_3sgn\left( e_{x_{1}}\right) +{\dot{\varphi }} \end{array} \right\} \end{aligned}$$

Now, the STC can be designed by replacing the states with their estimates using the HOSMO. Considered the same sliding surface mentioned in Eq. (14), the design of HOSMO based STC is as follows:

26 $$\begin{aligned} {\dot{{\hat{S}}}}=\sigma {\dot{e}}+\ddot{e}=\sigma \left( {{\dot{V}}}_{ref}-{\dot{{\hat{V}}}}_{pv}\right) +{{\ddot{V}}}_{ref}-{\ddot{{\hat{V}}}}_{pv} \end{aligned}$$

The time derivative of the sliding surface can be derived from Eq. (21) and expressed as follows:

27 $$\begin{aligned} {\dot{{\hat{S}}}}= & {} \sigma \left( {{\dot{V}}}_{ref}-{\hat{I}}_{L}-u-z_{1}\right) +{{\ddot{V}}}_{ref}-{\ddot{{\hat{V}}}}_{pv} \end{aligned}$$

28 $$\begin{aligned}= & {} \sigma \left( {{\dot{V}}}_{ref}-{\hat{I}}_{L}-u-l_1{\left| e_{x_{1}}\right| }^{\frac{2}{3}}sign\left( e_{x_{1}}\right) \right) +{{\ddot{V}}}_{ref}-{\ddot{{\hat{V}}}}_{pv} \end{aligned}$$

To derive a continuous control input $u_{con}$, it is assumed as the time derivative of the sliding surface is zero $( \dot{{\hat{S}}}=0 )$ at a finite time. Now, the control input becomes as follows:

29 $$\begin{aligned} u_{con}=-\frac{1}{\sigma }\left( {{\ddot{V}}}_{ref}-{\ddot{{\hat{V}}}}_{pv}\right) + {{\dot{V}}}_{ref}-{\hat{I}}_{L}-u-l_1{\left| e_{x_{1}}\right| }^{\frac{2}{3}}sign\left( e_{x_{1}}\right) \end{aligned}$$

To establish the sliding surface $( {\hat{S}})$ from input $u_{con}$, the Eq. (29) is substituted in Eq. (28). Then the ${{\hat{S}}}= 0$, indicates the asymptotic stability of *e* and ${\dot{e}}$^38^. Now, the final control signal with continuous and discontinuous modes becomes as follows:

30 $$\begin{aligned} \begin{aligned} u(t)&= -\sigma {{\dot{V}}}_{ref}+{\hat{V}}_{pv}+{\hat{I}}_{L}-{{\ddot{V}}}_{ref}-l_1{\left| e_{x_{1}}\right| }^{\frac{2}{3}}sign\left( e_{x_{1}}\right) \\&\quad -k_{1}\vert {\hat{S}}\vert ^{\frac{1}{2}}-\int ^{t}_{0} k_{2}sgn({\hat{S}}) d\tau \end{aligned} \end{aligned}$$

where, $k_{1}>0$ and $k_{2}>0$. The final closed-loop error dynamics are written as follows:

31 $$\begin{aligned} \Xi _{1}{:}\left\{ \begin{matrix} {{\dot{e}}}_{x_{1}}= &{} -l_1{\left| e_{x_{1}}\right| }^{\frac{2}{3}}sgn\left( e_{x_{1}}\right) +e_{x_{2}} \\ {{\dot{e}}}_{x_{2}}= &{} -l_2{\left| e_{x_{1}}\right| }^{\frac{1}{3}}sgn\left( e_{x_{1}}\right) +e_{x_{3}} \\ {{\dot{e}}}_{x_{3}}= &{} -l_3sgn\left( e_{x_{1}}\right) +{\dot{\varphi }} \end{matrix} \right. \ \end{aligned}$$

The stability of error $\Xi _{1}$ can be proved along the lines of^42^ and^37^.

**Figure 5:**
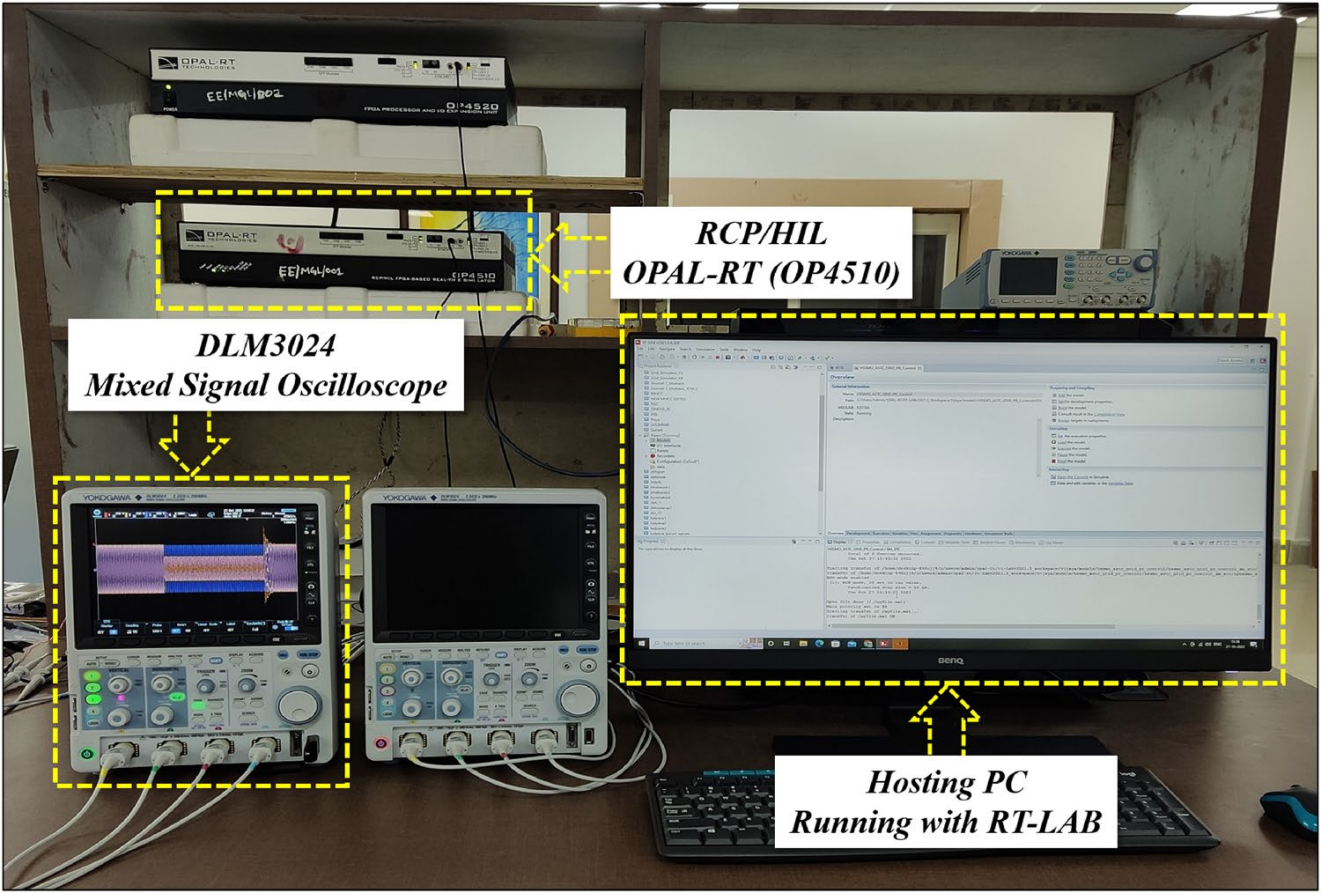
Real-time simulator setup for the proposed HOSMO based STC using OPAL-RT.

## Implementation of the proposed system on OPAL-RT

The real-time characteristics of the proposed microgrid model is evaluated by using OPAL-RT, which is a real-time simulator. The OPAL-RT platform seamlessly integrates with various programming environments, providing a complete range of rapid control prototyping solutions for iterating and testing, fast design, decreased development risks, time, cost, and control methodologies. The OPAL-RT has a wide range of applications in electrical systems, aerospace, electric vehicles, and more. Its console panel facilitates code creation and an interactive interface, enabling online parameter changes akin to a physical real-time system. However, real-time simulation often demands robust computing hardware, contributing to implementation costs. Moreover, integrating OPAL-RT with existing systems or workflows may present challenges, including compatibility issues and customization requirements. Additionally, real-time simulation typically requires powerful computing hardware, adding to the overall cost of implementation. Integration with existing systems or workflows may also pose challenges, particularly regarding compatibility issues or customization requirements^43^. Implementing microgrid systems in real-time with OPAL-RT presents several challenges. Ensuring the accuracy of simulation models to reflect real-world behaviour is paramount, as any disparities can lead to inaccurate results. Managing the large volume of data generated during real-time simulation also poses a challenge, necessitating effective data management strategies. Additionally, encountering algebraic loop errors can be a common issue, requiring careful debugging and adjustment of model equations to resolve^44^.

To conduct an OPAL-RT experiment, the system needs to execute in RT-LAB software as follows:

- Before running in the simulator, the proposed microgrid model is executed for a fixed time step in MATLAB/SIMULINK according to the model requirement and hardware capability
- After that, the considered model is divided into two groups (a) SM_Master block and (b) SM_Console block
- The inputs and outputs of the system must go through the *opcomm* blocks
- And finally, with RT-LAB, the developed microgrid model is executed by converting the SIMULINK model into C code by using *intel xeon-2667 V3 @2.32 GHZ* processor and *Xilink FPGA Knight-7.325T*

The outputs are visualized in the user interface window in the hosting computer. These outputs under different test conditions are collected from the OPAL-RT (OP4510) by using the mixed signal oscilloscope (DLM3024) with the help of analog outputs (*opctrl* block) as shown in Fig. 5. The real-time accuracy and details of OP4510 Simulator are reported in^45^.

**Figure 6:**
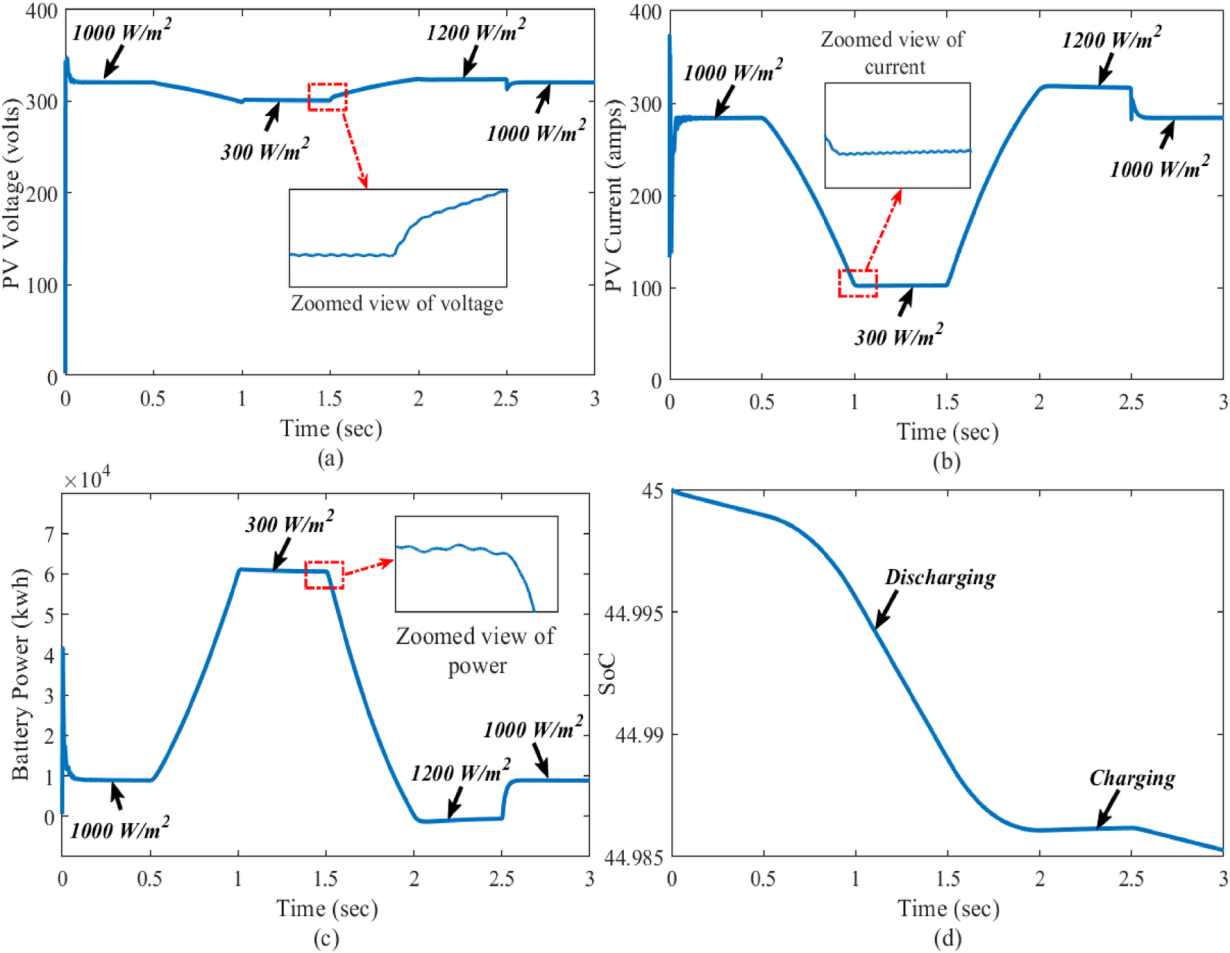
Closed-loop simulations of the system in the presence of change in irradiations; where the subfigures (**a**–**c**) and represents PV voltage, PV current, battery power, and state of the charge, respectively.

**Figure 7:**
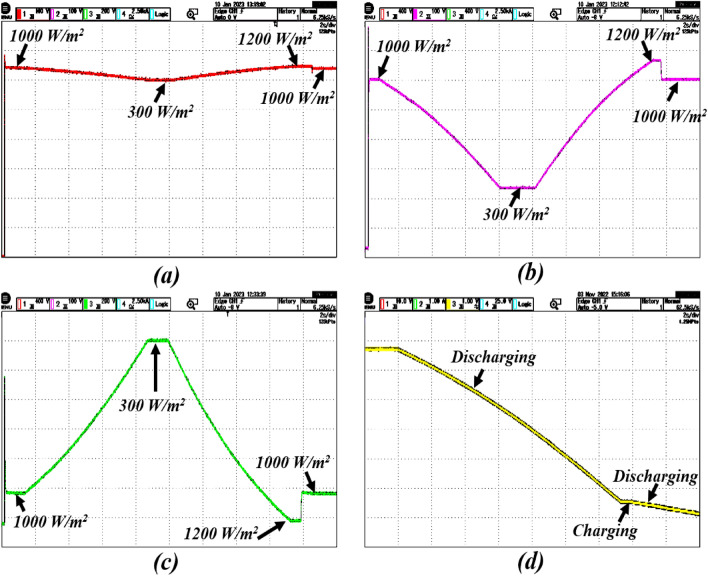
Real-time results of the system in the presence of change in irradiations; where the subfigures (**a**–**c**) and represents PV voltage, PV current, battery power, and state of the charge, respectively.

**Figure 8:**
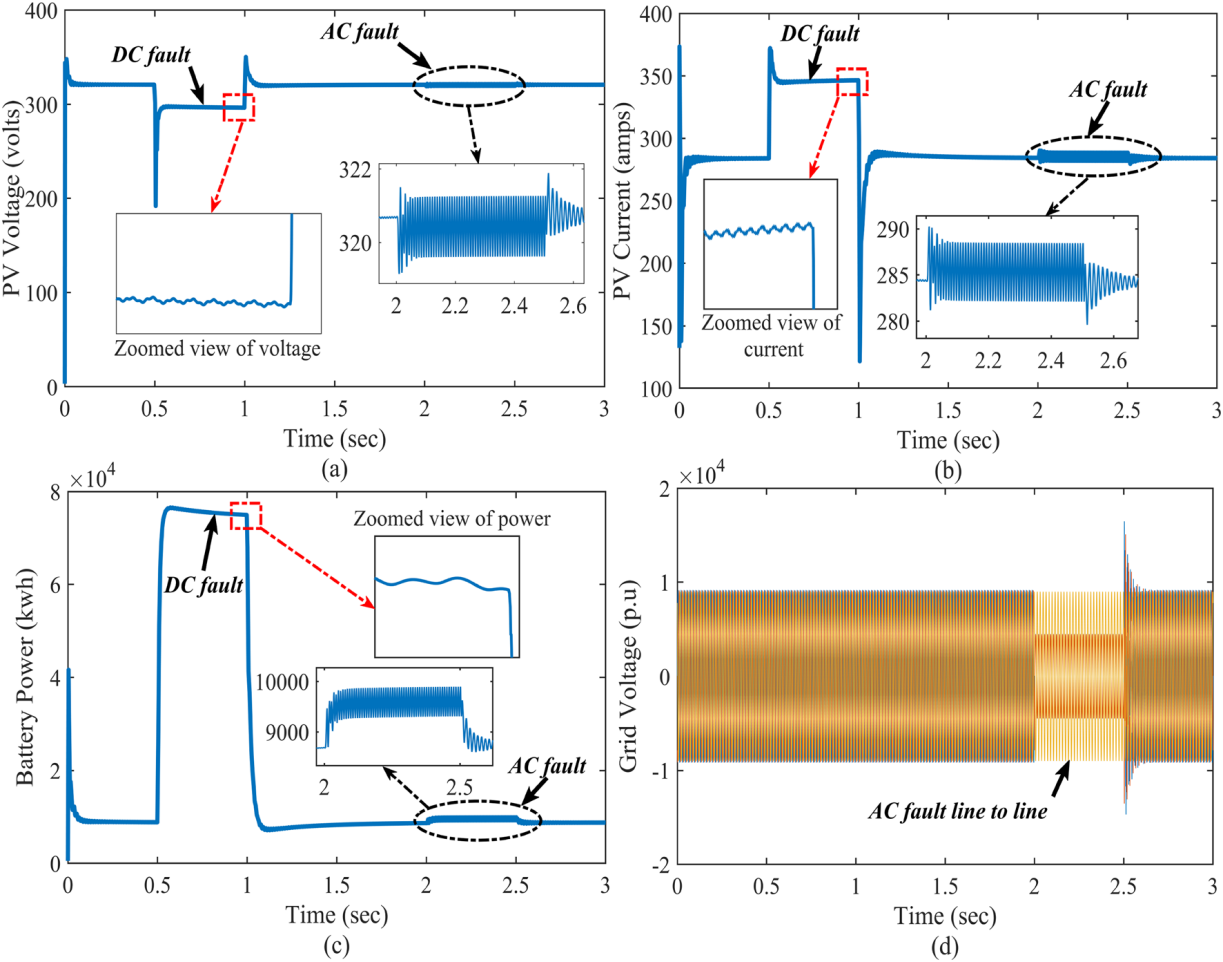
Closed-loop simulations of the system with DC and AC faults; where the subfigures (**a**–**c**) represents PV voltage, PV current, battery power, and grid voltage, respectively.

## Results and discussion

Various simulation test condition results are reported to demonstrate the efficacy of the proposed control scheme to obtain MPP in the case of a grid-connected PV array. The following test scenarios are simulated and tested to verify the controller’s effectiveness and robustness against the unpredictable parametric variation and dynamic fault response. The simulation and real-time results were carried out by using MATLAB/Simulink software and OPAL-RT simulator. Advanced state estimation techniques like EKF and STO are considered for comparative study to analyze the proposed HOSMO performance and STC.

### Case-1: dynamic change in solar irradiations

In this case, the performance of the proposed control is verified under the dynamic change in solar irradiations with constant temperature and load. The solar irradiation is varied within the range from 1000 to 300 w/m$^{2}$ and 300 to 1200 w/m$^{2}$. To reach the power demand of the grid and DC load, the battery also delivers the power as shown in Fig. 6. The real-time performance of the grid with the solar irradiation change is shown in Fig. 7. Here, the reference voltage of the PV array is 360 volts, which is also the MPP of standard testing conditions of the PV array. There is a gradual effect on the output of the PV array due to the change in solar irradiation. Here, inductor current is estimating through HOSMO on partially shaded condition. This component helps to estimate the solar irradiance on the PV modules, taking into account the partial shading conditions^46,47^. A constructed HOSMO-STC for precise determination of the optimal power areas of the PV array. This control network is accurately identify the optimal power areas of the PV array under various partial shading patterns. The proposed control system reacts quickly to stabilize the system, while the other controllers take a longer time to establish finite time stability. Figures 6 and 7 clearly illustrate that the control scheme provides robust stability with fast convergence with the drastic change in solar irradiation to PV output in MATLAB/Simulink and real-time conditions.

### Case-2: response of dynamical faults

**Figure 9:**
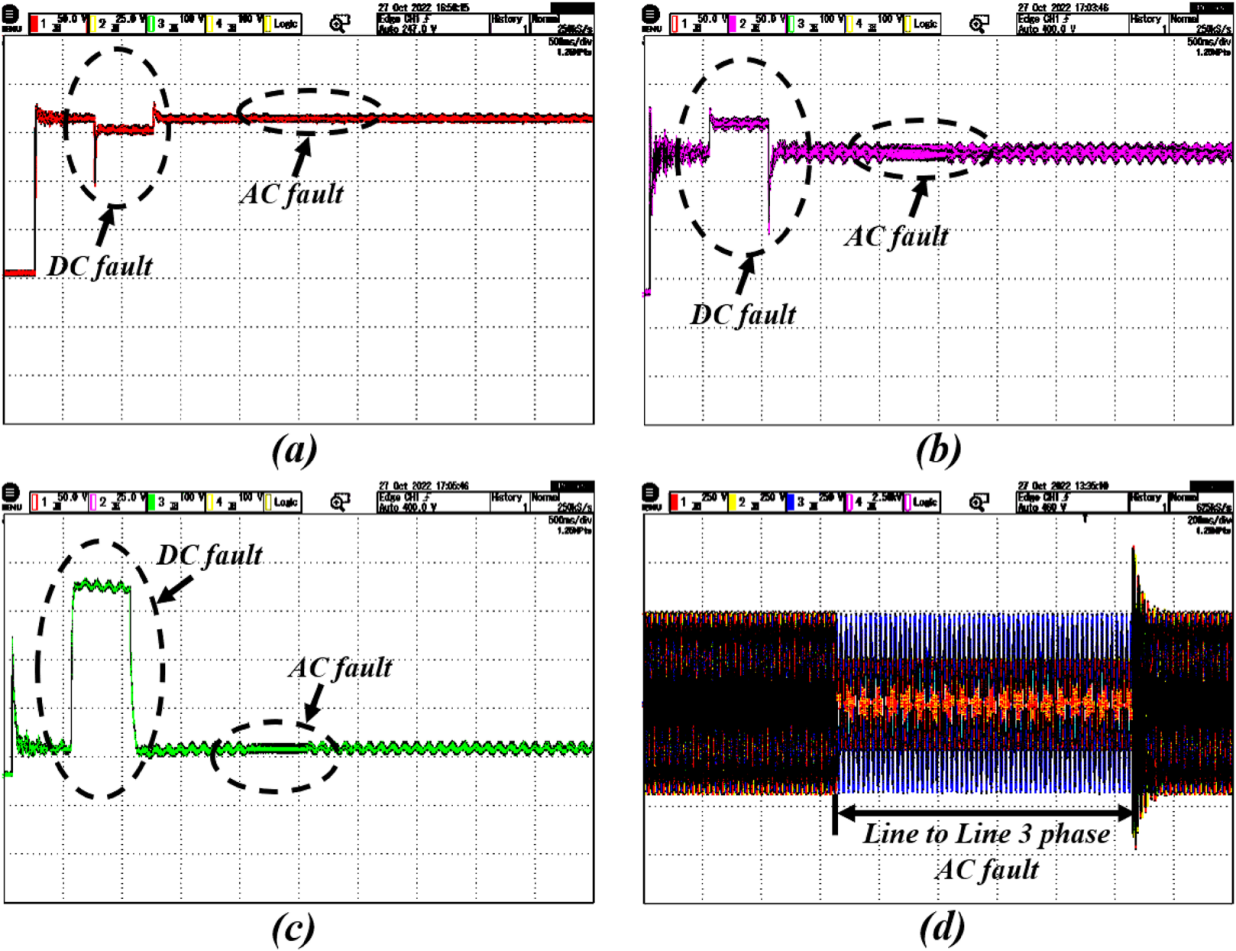
Real-time results of the system with DC and AC faults; where the subfigures (**a**–**c**) represents PV voltage, PV current, battery power, and grid voltage, respectively.

In reality, there is a chance to faults occur due to rapid variations present in the system. In this case, the DC and AC faults are created knowingly in the Simulink model to verify the control system’s real-time performance (in OPAL-RT) under the dynamical faults. Here, the DC fault is created during t = 0.5 to 1 s, and a line-to-line AC fault is created between t = 2 and 2.5 s. Figures 8 and 9 reflect the impact of the DC and AC faults on PV voltage, current, battery power, and grid voltage. Here, the proposed control system provides a strong control signal to stand by with PV output and DC–DC boost converter output when sudden faults occur.

### Case-3: performance justification during islanding and resynchronization

In grid-connected mode, there is a risk of power imbalance owing to the duration of time when the grid supply is unavailable. Because bidirectional power is not attainable in that situation, the utility is disconnected from the PV-Battery system for that time. In the process of resynchronizing islanded (IS) mode to grid-connected (GC) mode, a static switch plays a crucial role in facilitating the seamless transition. Initially, the system carefully monitors grid conditions, including frequency, voltage, and phase angle, using sensors and monitoring devices. Upon confirming that grid conditions are within acceptable parameters, the static switch is activated to connect the system to the grid. This activation enables the system to adjust its parameters, such as frequency, voltage, and phase angle, to synchronize with the grid’s parameters. Concurrently, the static switch ensures a smooth transition by providing a controlled connection between the system and the grid, minimizing disruptions and potential damage to equipment. Once synchronization is achieved and verified, the static switch remains engaged, allowing the system to operate in grid-connected mode efficiently. Therefore, through the strategic use of a static switch, the resynchronization process is facilitated, ensuring a safe and reliable transition from islanded mode to grid-connected operation^48^.

**Figure 10:**
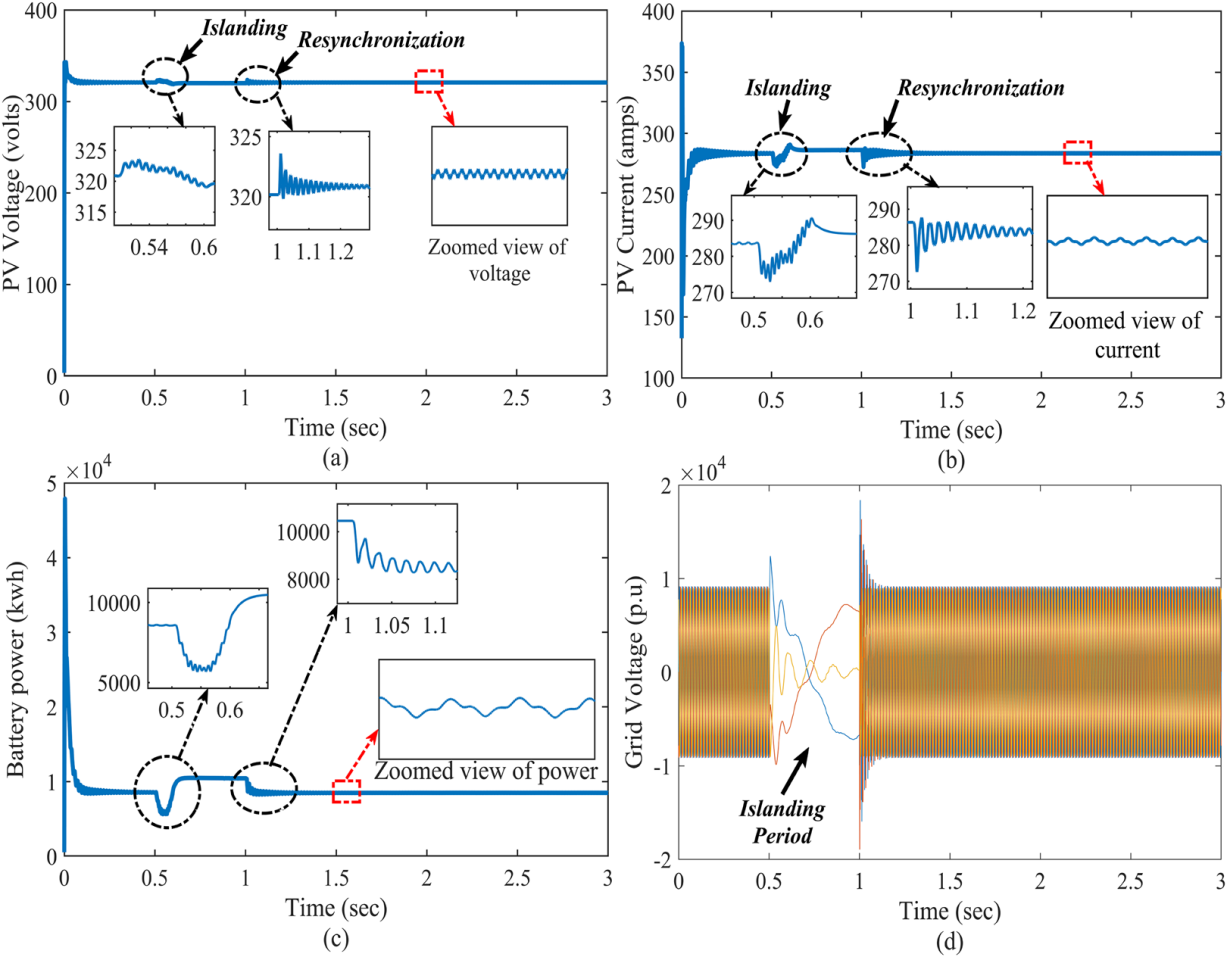
Simulation results of the system with islanding and resynchronization; where the subfigures (**a**–**c**) represents PV voltage, PV current, battery power, and grid voltage, respectively.

**Figure 11:**
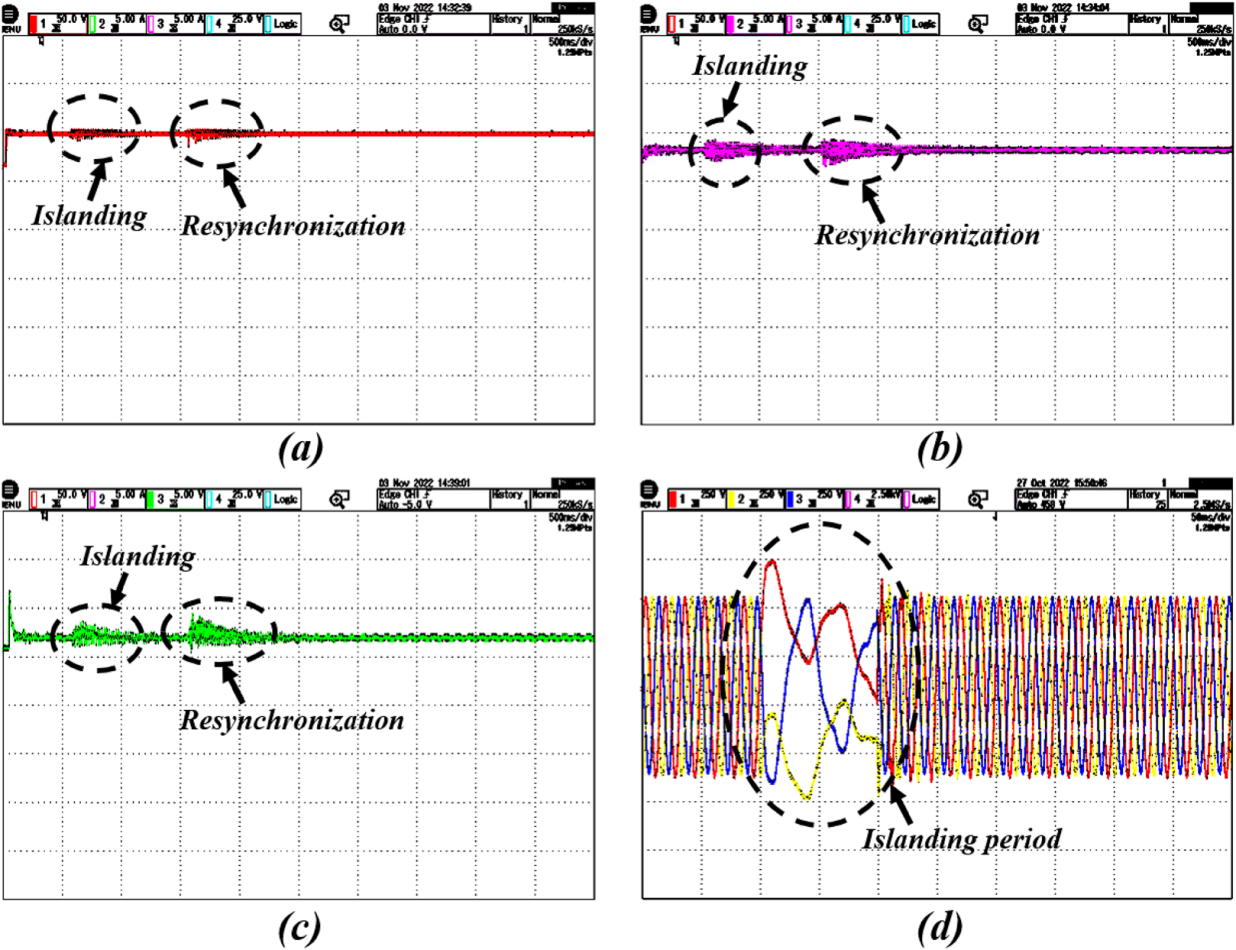
Real-time results of the system with islanding and resynchronization; where the subfigures (**a**–**c**) represents PV voltage, PV current, battery power and grid voltage, respectively.

In this case, to verify the performance of the overall system, the PV-Battery system is made intentionally islanded from the grid at t = 0.5–1 s. During that time, the system is operated in standalone mode, and at the same time desired power is provided by the BES. During this period, the battery is in discharging mode. The system is resynchronized to the grid after reaching its normal position at t = 1 s. The islanding and resynchronizing with the grid at the point of common coupling (PCC) are done by a static transfer switch. The impact of the islanding and resynchronization transitions during this period on the PV-Battery system in real-time is evaluated and illustrated in Figs. 10 and 11.

**Figure 12:**
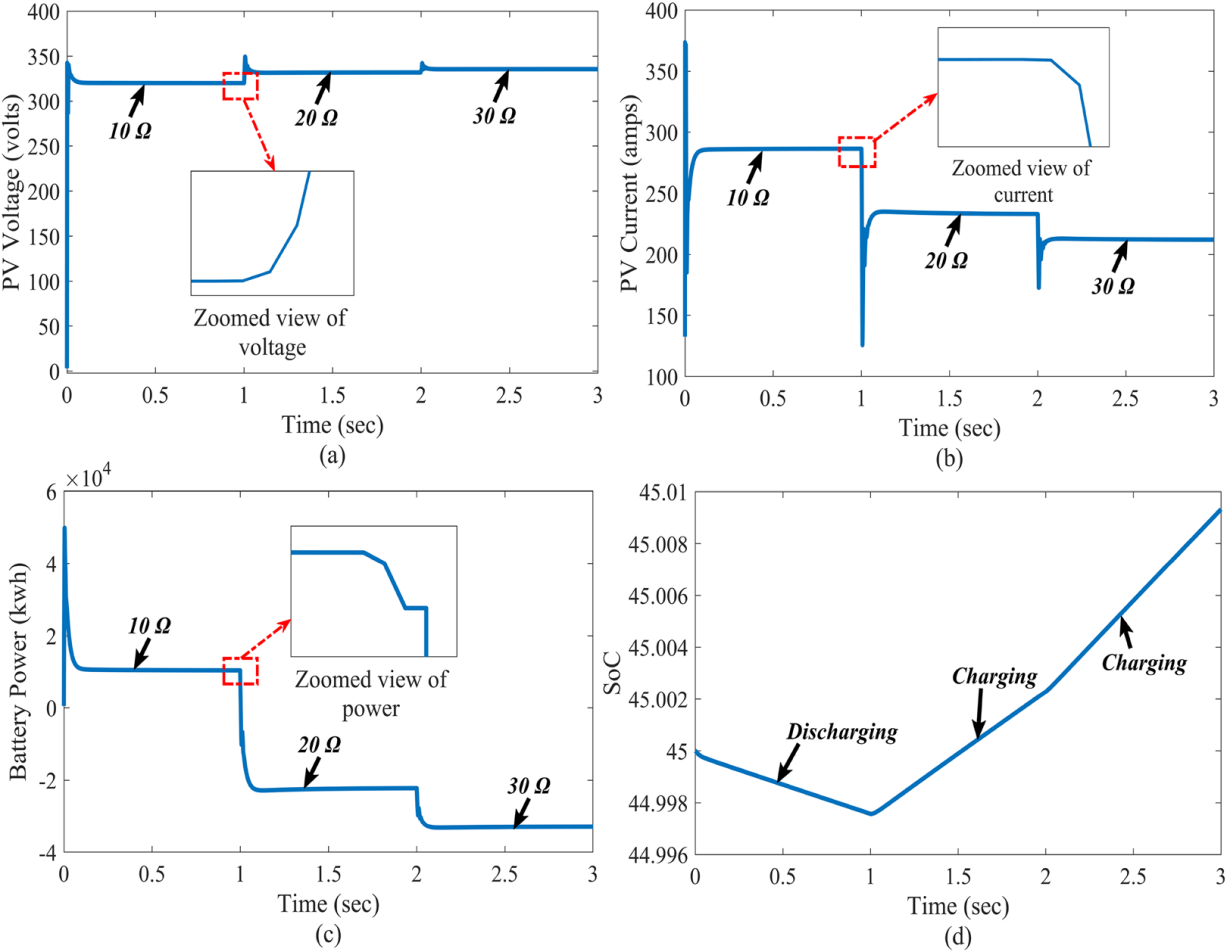
Simulation results of the system with varying load resistance; where the subfigures (**a**–**c**) represents PV voltage, PV current, battery power, and state of the charge, respectively.

**Figure 13:**
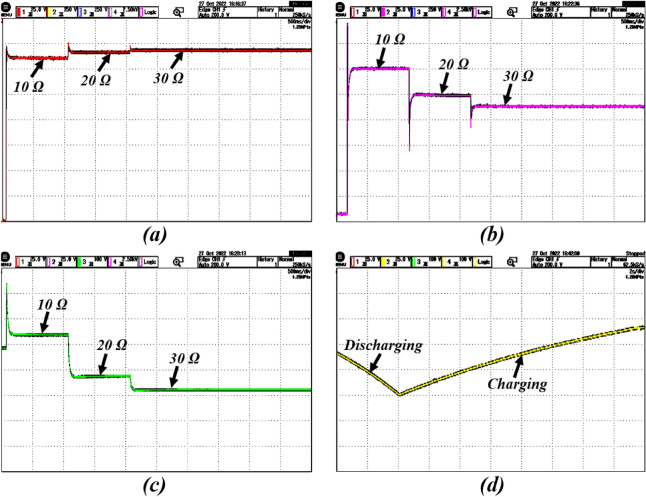
Real-time results of the system with varying load resistance; where the subfigures (**a**–**c**) represents PV voltage, PV current, battery power, and state of the charge, respectively.

### Case-4: response due to DC load change

Very often, there is a rapid change in load patterns in real-time applications. To validate the control system performance under sudden load changes, different resistive loads are connected at the DC side of the system in the test system. The load resistances are taken as $10\Omega$ to $20\Omega$ and $30\Omega$, respectively, to conduct experiments under different conditions. Figures 12 and  13 show the influence of DC load variation on the PV battery system. The results indicate the proposed control super-twisting scheme attained the MPP under different load conditions. Even if the AC Load will change the DC link capacitor will protect the DC bus voltage from the disturbance. Further, there is no change in DC Voltage. In this situation also proposed system is maintaining stable voltage.

### Case-5: comparison of the proposed scheme with extended Kalman filter and super-twisting observer

**Figure 14:**
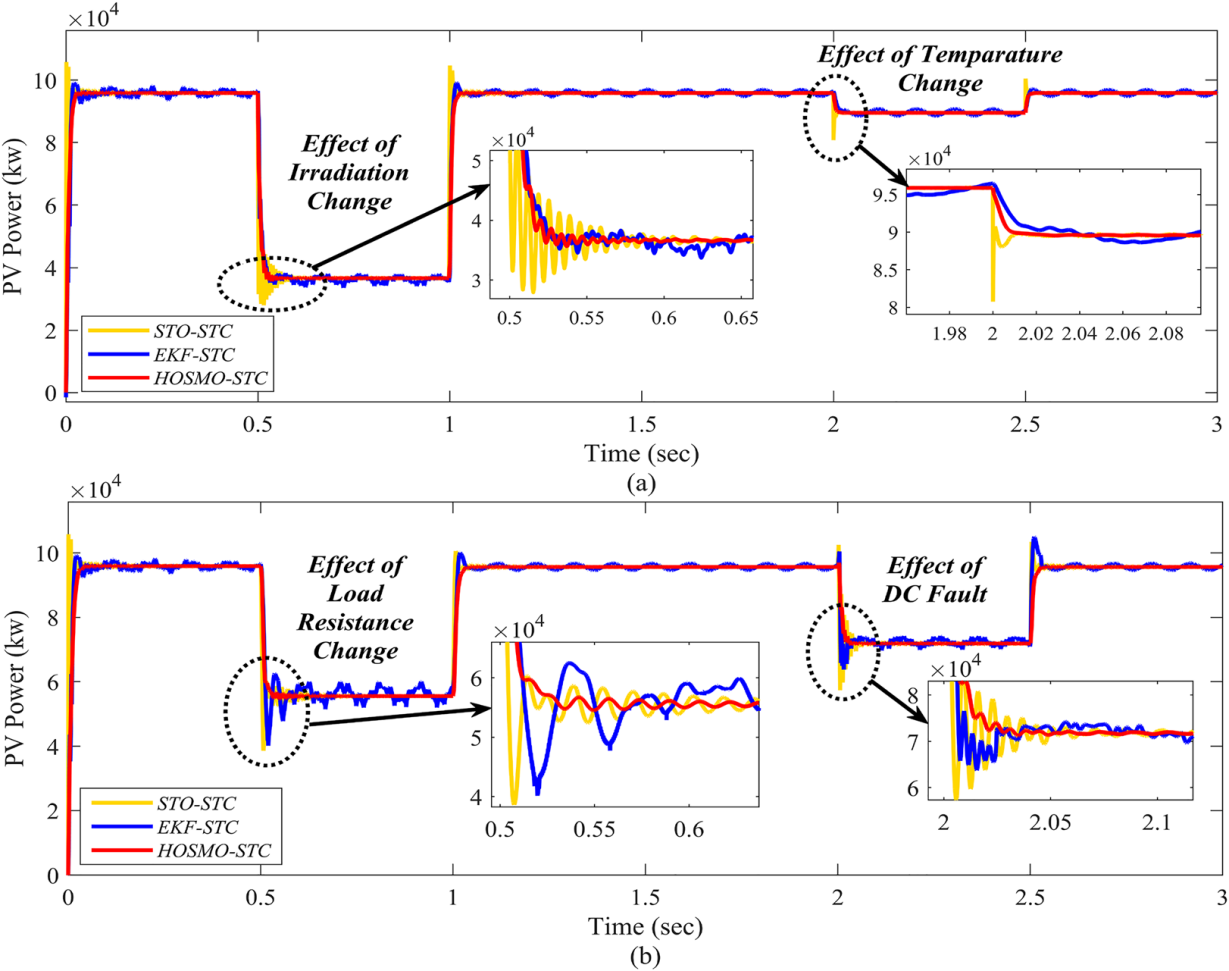
Comparison with EKF and STO; the subfigures (**a**) and (**b**) represent effect of irradiation and temperature change and the effect of load resistance change and DC fault, respectively.

In this section, the proposed controller’s performance is evaluated and demonstrated with different cases like irradiation, temperature, DC load resistance variations, and the effect of DC fault. The obtained comparative results in comparison to the advanced state estimation techniques like EKF and STO. The design procedure of the EKF is adopted from^49^. The EKF has been designed with the linearized state matrix. The linearization is done by using the discrete-time small-signal approximation presented in^50^. Likewise, the STO is developed based on the procedure presented in^23^. Figure 14 reflects the enhanced performance of the proposed control system strategy, and the results show a substantial improvement compared to the EKF and STO. The steady state response of the proposed controller is analyzed from the zoomed views of Fig. 14 and indicates the system performance. From all test cases, the zoomed views of Figs. 6, 8, 10,  12 and 14 demonstrate that the proposed controller effectively reduces chattering and performs better in comparison. It provides a significant enhancement in real-time with MPPT control for the grid-connected PV-Battery system.

Finally, the effect of different inductance and capacitance values on the responses of inductor current and capacitor voltage are provided in the supplementary material S1.

## Conclusions

A super-twisting MPPT controller based on higher order sliding mode observer is proposed for the grid-connected PV-Battery system. The proposed control strategy ensures maximum power during the operation with a limited number of sensors. The proposed super-twisting controller ensures finite-time stability of the overall closed microgrid, where HOSMO ensures accurate estimation of the inductor current. The simulation and real-time results are presented for verification and to justify the feasibility of the real-time application of the proposed control strategy. Looking at the practical application, different test cases are considered, which often occur in PV-Battery based grid-connected systems like change in solar irradiations, dynamical faults, grid-connected mode to islanding, and DC load variations.With the proposed system, there is a continuous power supply to the DC load in the presence of nonlinearity of the generating source in PV. Thus, the efficiency of the system is reaching almost $95.039\%$. Achieving exact finite-time stabilization (dynamic collapse) and precise disturbance compensation for Single-Input Single-Output (SISO) systems with arbitrary relative degree is a challenging task to the proposed controller. This will model in future with advanced technologies. Moreover, large number of DGs integrated with utility grid under the partial shading conditions will be studied. The real-time performance of the proposed HOSMO-STC strategy for MPPT control is validated using an OPAL-RT (OP5700) simulator. The results reveal that the proposed control system shows superior performance and stability for various test cases. The proposed control strategy’s efficacy has been observed and compared with other state estimation techniques like extended Kalman filter and STO.

### Supplementary Information

Supplementary Information.

## Data Availability

All data generated or analyzed during this study are included in this published article.
