# Supplementary material for: Super-twisting MPPT control for grid-connected PV/battery system using higher order sliding mode observer
Source: Sci Rep. 2024 Jul 18;14:16597. doi: 10.1038/s41598-024-67083-w (PMC11258299; doi:10.1038/s41598-024-67083-w)
Supplement: Supplementary file 1 — Supplementary Information. [file 41598_2024_67083_MOESM1_ESM.docx]

**Supplementary Material**

Accurate values of inductance (L) and capacitance (C) are critical in boost converters to ensure stable operation, efficiency optimization, and prolonged component lifespan. By properly sizing these components, energy losses can be minimized, electromagnetic interference reduced, and control loop design enhanced, resulting in reliable performance across diverse operating conditions. The L and C values of proposed system has been decided according to the response presented in Fig. S1.


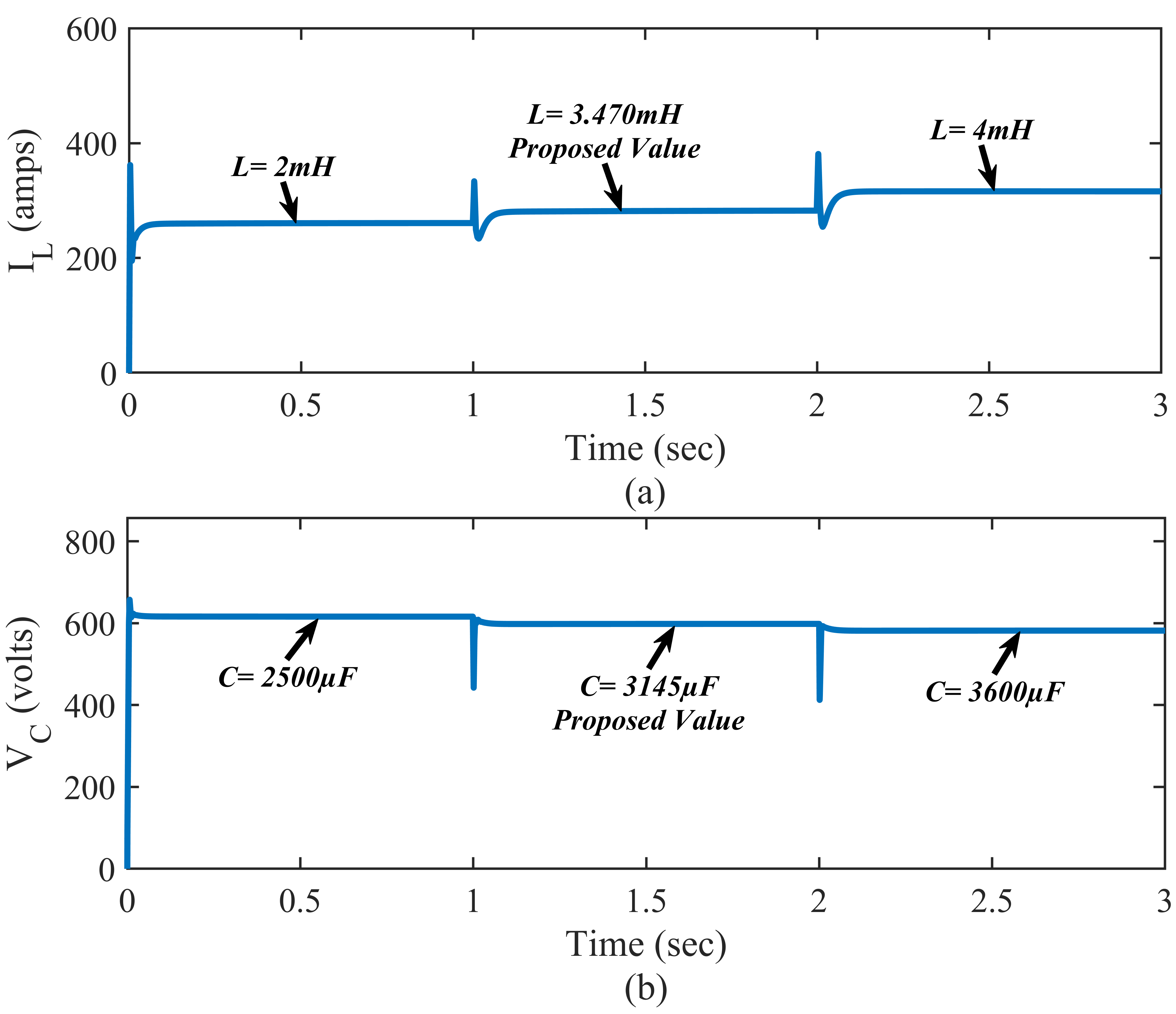


**Fig. S1. Response of the (a) inductor current and (b) capacitor voltage for different inductance and capacitance values.**
